# Real-time qPCR improves meningitis pathogen detection in invasive bacterial-vaccine preventable disease surveillance in Fiji

**DOI:** 10.1038/srep39784

**Published:** 2016-12-23

**Authors:** Eileen M. Dunne, Silivia Mantanitobua, Shalini P. Singh, Rita Reyburn, Evelyn Tuivaga, Eric Rafai, Lisi Tikoduadua, Barbara Porter, Catherine Satzke, Janet E. Strachan, Kimberly K. Fox, Kylie M. Jenkins, Adam Jenney, Silo Baro, E. Kim Mulholland, Mike Kama, Fiona M. Russell

**Affiliations:** 1 Murdoch Childrens Research Institute, Infection and Immunity, Parkville, 3052, Australia; 2 Ministry of Health & Medical Services, Suva, Fiji; 3 The University of Melbourne, Department of Microbiology and Immunology, Peter Doherty Institute for Infection and Immunity, Melbourne, 3000, Australia; 4 The University of Melbourne, Department of Paediatrics, Parkville, 3052, Australia; 5 The University of Melbourne, Microbiological Diagnostic Unit Public Health Laboratory, Melbourne, 3000, Australia; 6 World Health Organization Regional Office for the Western Pacific, Manila, Philippines; 7 Fiji Health Sector Support Program, Suva, Fiji; 8 Fiji National University, Department of Medical Science, College of Medicine, Nursing and Health Sciences, Suva, Fiji; 9 London School of Hygiene and Tropical Medicine, Department of Infectious Disease Epidemiology, London, WC1E 7HT, UK

## Abstract

As part of the World Health Organization Invasive Bacterial-Vaccine Preventable Diseases (IB-VPD) surveillance in Suva, Fiji, cerebrospinal fluid (CSF) samples from suspected meningitis patients of all ages were examined by traditional methods (culture, Gram stain, and latex agglutination for bacterial antigen) and qPCR for *Streptococcus pneumoniae, Neisseria meningitidis*, and *Haemophilus influenzae*. Of 266 samples tested, pathogens were identified in 47 (17.7%). *S. pneumoniae* was the most common pathogen detected (n = 17) followed by *N. meningitidis* (n = 13). The use of qPCR significantly increased detection of IB-VPD pathogens (P = 0.0001): of 35 samples that were qPCR positive for *S. pneumoniae, N. meningitidis*, and *H. influenzae*, only 10 were culture positive. This was particularly relevant for *N. meningitidis*, as only 1/13 cases was culture positive. Molecular serotyping by microarray was used to determine pneumococcal serotypes from 9 of 16 (56%) of samples using DNA directly extracted from CSF specimens. Results indicate that qPCR significantly increases detection of *S. pneumoniae, N. meningitidis*, and *H. influenzae* in CSF, and that application of molecular diagnostics is a feasible way to enhance local and global surveillance for IB-VPD.

Bacterial meningitis is a severe infection that results in high rates of morbidity and mortality worldwide^1^. Vaccines against the three most common bacterial causes of meningitis, *Streptococcus pneumoniae, Neisseria meningitidis*, and *Haemophilus influenzae* type b (Hib) are available, but do not protect against all bacterial serotypes/serogroups, and are not widely available in some resource-limited countries. Surveillance of bacterial meningitis provides useful information on the predominant causes of meningitis in a population, is essential for outbreak detection, and can help to monitor changes in meningitis rates and aetiology following vaccine introduction^2^,^3^,^4^.

The aetiologic agents of bacterial meningitis are typically identified from cerebrospinal fluid (CSF) by culture, Gram stain and latex agglutination testing^5^. However, sensitivity of these methods is limited, particularly when patients receive antibiotic treatment prior to sample collection^5^. The use of molecular methods such as real-time quantitative PCR (qPCR) can improve sensitivity for detection of meningitis pathogens^6^,^7^,^8^,^9^. The World Health Organization (WHO) recommends qPCR testing of *S. pneumoniae, N. meningitidis*, and *H. influenzae* from CSF of suspected meningitis cases as part of Invasive Bacterial Vaccine Preventable Diseases (IB-VPD) surveillance^10^.

Fiji is an island nation in the South Pacific with an estimated population of 869,458 (http://www.statsfiji.gov.fj/). Hib vaccine was introduced into the routine immunisation schedule in 1997. Meningococcal vaccine has not been introduced. The 10 valent Pneumococcal Conjugate Vaccine (PCV10, Synflorix^®^) was added to Fiji’s national infant immunisation program in 2012. As part of the New Vaccine Evaluation Project, IB-VPD surveillance was established at the Colonial War Memorial Hospital (CWMH) in Fiji’s capital, Suva, and qPCR testing for common bacterial causes of meningitis established at the Fiji Centre for Communicable Disease Control (FCCDC). Here, we present results from examination of 266 CSF samples by traditional microbiological methods and qPCR for *S. pneumoniae, N. meningitidis*, and *H. influenzae*. The aims of this study were to identify the aetiologic agents causing meningitis in the years immediately following PCV introduction, and to examine whether the use of qPCR improved detection of *S. pneumoniae, N. meningitidis*, and *H. influenzae* compared to traditional microbiological approaches.

## Methods

### Sample collection and processing

CSF samples were obtained from patients of all ages at the CWMH in Suva, Fiji from November 2012 to May 2016. According to routine clinical care, lumbar punctures were performed on patients with suspected acute meningitis (except in those with contra-indications such as signs of raised intracranial pressure), defined as sudden onset of fever >38 °C and one of the following signs: neck stiffness, altered consciousness with no other alternative diagnosis, or other meningeal sign. All methods were carried out in accordance with relevant guidelines and regulations. Protocols and procedures were approved by the Fijian Ministry of Health and Medical Services as part of a Research Agreement. In accordance with CWMH and Fijian Ministry of Health and Medical Services policies, individual informed consent was not required as sample collection and testing were conducted as part of routine diagnostics and public health surveillance.

CSF samples were collected and processed immediately upon receipt by the CWMH microbiology laboratory using methods based on WHO recommendations^10^,^11^. In brief, samples were examined macroscopically prior to centrifugation. Glucose levels were determined by using the BS-800 or BS-2000 chemistry analyzer (Mindray) and protein levels were determined by spectrophotometer (GENESYS 10 S UV-Vis spectrophotometer) at the CWMH Biochemistry laboratory. Differential cell counts and Gram staining were performed by light microscopy. Direct antigen testing for *H. influenzae* type b, *S. pneumoniae, N. meningitidis* groups A, C, Y, W135, *N. meningitidis* group B, *E. coli* K1, and group B streptococcus was performed using a Wellcogen Bacterial Antigen Rapid Latex Agglutination Test (ThermoFisher Scientific). Direct antigen testing was only performed on 107/266 (40%) of samples as the latex testing kits were not always in stock. Samples were cultured on human blood agar, chocolate blood agar, and MacConkey agar, and incubated at 37 °C with 5% CO_2_ for 48 h. Human blood agar was used as a substitute for sheep/horse blood agar due to inconsistent availability of animal blood from local suppliers. Any resultant growth was subject to identification by standard methods^11^.

Remaining CSF was held at room temperature for one week and monitored for cloudiness (indicative of bacterial growth), and all specimens transferred to the FCCDC for qPCR analysis. Following a review by the WHO Regional Office in March of 2015, CSF samples were temporarily held at 4 °C rather than room temperature. At the FCCDC, samples were stored at −80 °C until use.

### Nucleic acid extraction and qPCR

Samples were thawed and DNA extraction and qPCR performed according to WHO guidelines^10^. In brief, samples were thawed and 200 μl used for DNA extraction. If less than 200 μl was available, all remaining volume was used, and for samples with no visible liquid the specimen tube was rinsed with 200 μl of TE buffer. CSF was centrifuged at 7300 × g for 10 min, and the pellet subjected to bacterial lysis performed by enzymatic digest in lysozyme (0.4 g/ml) and mutanolysin (0.075 mg/ml) in TE buffer incubated at 37 °C for 1 h followed by the addition of lysis buffer and Proteinase K and incubation for a further 30 min at 56 °C. Lysis was completed and DNA extraction performed using the QIAamp DNA Mini Kit (Qiagen) according to the manufacturer’s instructions, and DNA eluted in 50 μl. For qPCR, 2 μl of template was used in 25 μl singleplex reactions containing iTaq Universal Probes Supermix (Bio-Rad) and primers and probes specific for *lytA (S. pneumoniae*), *sodC (N. meningitidis*), and *hpd (H. influenzae*)^10^. Samples were run on a Bio-Rad CFX96 Thermal Cycler (95 °C for 3 min followed by 40 cycles of 95 °C for 5 sec and 60 °C for 20 sec). Samples with a Ct value <35 were considered positive, >40 negative, and 35–40 equivocal, unless otherwise specified. Equivocal samples were diluted 1:4 and 1:10 to dilute possible inhibitors and retested. Although the real-time PCR assay was not fully quantitative as a standard curve was not run, the abbreviation qPCR is used for consistency with other published studies.

### Capsular typing

Any samples positive for *hpd* were further examined by *bcsB* qPCR, which specifically detects *H. influenzae* type b^10^. Pneumococcal molecular serotyping by microarray (Senti-SPv1.5, BUGS Bioscience) was conducted on *lytA* positive DNA extracted from CSF samples, and conventional serotyping by latex agglutination was performed on pneumococcal isolates at the Murdoch Childrens Research Institute as previously described^12^,^13^. Meningococcal typing was performed at the WHO Invasive Bacterial Vaccine Preventable Diseases Regional Reference Laboratory at The University of Melbourne by latex agglutination on isolates^10^. Meningococcal serogrouping by qPCR was conducted using DNA extracted from CSF using primers and probes for *sacB, synD, synE, synG, xcbB*, and *synF* for serogroups A, B, C, W135, X, and Y, respectively, as previously described^10^.

### Statistical analysis

Statistical analyses were performed using GraphPad Prism version 5.04 for Windows (GraphPad Software, San Diego, CA, USA). Any bacterial species identified by culture, qPCR, and/or direct antigen testing was considered a positive identification. Fisher’s exact test was used to compare categorical values. Datasets were tested for normal distribution using the D’Agostino-Pearson normality test. The unpaired t test and Mann-Whitney U test were used to examine for differences between normally and non-normally distributed datasets, respectively. All tests were two-tailed. P values < 0.05 were considered statistically significant.

## Results

### Patient characteristics and pathogen detection

**Figure 1:**
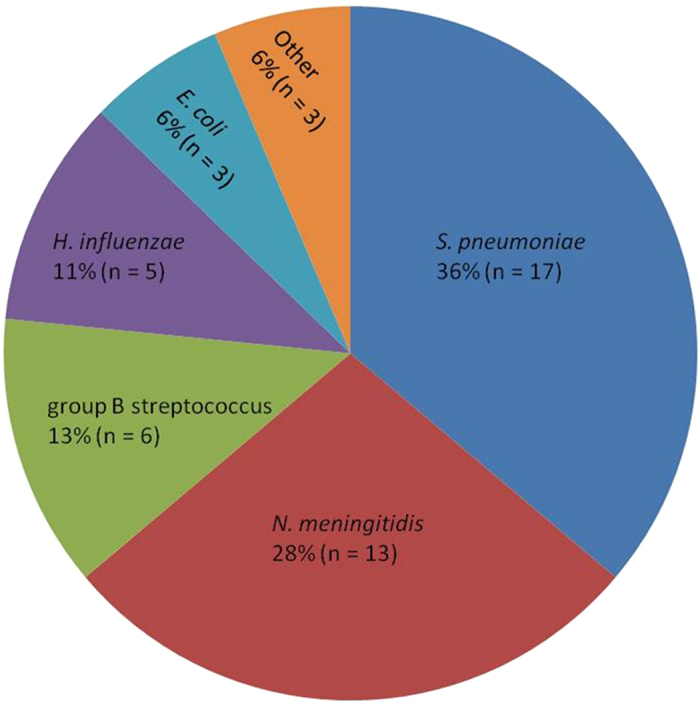
Aetiology of bacterial meningitis in Fiji (n = 47). Other includes *Klebsiella oxytoca, Acinetobacter baumannii*, and coagulase negative staphylococci (n = 1 each).

A total of 266 CSF samples were included in this study. Patient characteristics are summarised in Table 1. Three patients had two samples collected during their illness; both samples were included in the study. CSF samples were examined by traditional methods including culture, Gram stain, and direct antigen testing by latex agglutination as well as qPCR for detection of *S. pneumoniae, N. meningitidis*, and *H. influenzae*. A total of 47 (17.7%) samples had a specific aetiologic agent identified, with *S. pneumoniae* (n = 17), *N. meningitidis* (n = 13), and group B streptococcus (n = 6) most common (Fig. 1). Two samples with bacteria visible on Gram stain (one with Gram positive cocci and one with Gram positive diplococci), but negative by all other tests, were not included in aetiology results as species could not be definitively identified by Gram stain alone, although they were classified as bacterial positive samples. All six samples containing group B streptococcus were identified by direct antigen testing and were negative by culture and Gram stain. Two samples with qPCR Ct values in the equivocal range for *N. meningitidis* were classified as positive for the following reasons: low volumes of CSF available for DNA extraction (50 μl and 30 μl as opposed to the recommended 200 μl, which lowers DNA yield and can lead to higher Ct values), clinical signs and symptoms consistent with meningococcal meningitis, and follow-up qPCR for capsular typing gave similar Ct values for serogroup B. Of the 49 bacterial positive samples, 36 (73%) were from children between one month and five years of age. Aetiological agents identified by patient age group and clinical characteristics are shown in Table 1.

### CSF characteristics

CSF samples that tested positive for bacteria had lower levels of glucose (median 1.2 mmol/L; IQR 0.3, 2.8 mmol/L) compared to samples that tested negative (median 3.0 mmol/L; IQR 2.3, 3.8 mmol/L, P < 0.0001, Mann-Whitney test). Bacteria positive samples had higher levels of protein (median 962 mg/L; IQR 378, 1986 mg/L) compared to negative samples (median 360 mg/L; IQR 160, 795 mg/L, P < 0.0001, Mann-Whitney test). The majority of samples included in this study (242/266; 91%) had elevated white blood cell counts (>10 cells/mm^3^), an indicator of meningitis. The median white cell count for samples testing positive for bacteria was 240 × cells/mm^3^ (IQR 98, 1190 cells/mm^3^) compared to 32 cells/mm^3^ (IQR 14, 132 cells/mm^3^) for negative samples (P < 0.0001, Mann-Whitney test).

Of the 265 samples tested by qPCR, CSF volumes used for DNA extraction ranged from 0–200 μl (median 200 μl; IQR 106.5, 200 μl) with 160 (60%) of samples containing the recommended 200 μl volume of CSF available for extraction. There was no significant difference in the sample volumes of qPCR positive samples (median 181 μl; IQR 105, 200 μl) compared to qPCR negative samples (median 107 μl; IQR 107, 200 μl, P = 0.3, Mann-Whitney test) and the lowest sample volume that was qPCR positive was 0 μl (collection tube had no visible liquid and was rinsed with 200 μl buffer for extraction).

### Methods comparison

A comparison of detection methods for *S. pneumoniae, N. meningitidis*, and *H. influenzae* is shown in Table 2. Testing by qPCR identified an additional 23 cases of IB-VPD compared to culture alone (P < 0.0001, Fisher’s exact test). One sample that was culture positive for *S. pneumoniae* did not have qPCR performed. One sample that was positive for *N. meningitidis* by direct antigen testing and had Gram negative diplococci seen by microscopy was negative by qPCR; however, only one μl of CSF was available for DNA extraction. Only 17/35 (49%) of samples had direct antigen testing performed, as kits were not always in stock. Gram staining was performed for all except for two samples. When methods were assessed for sensitivity (excluding samples that were not tested), qPCR had a sensitivity of 97% (33/34), compared to 45% (9/20) for direct antigen testing and 29% (10/35) for culture. Although Gram stains do not provide definitive species identification, results were consistent with the identified species (Gram positive diplococci for *S. pneumoniae*, Gram negative diplococci for *N. meningitidis*, and Gram negative coccobacilli for *H. influenzae*) for 36% (12/33) samples.

### Capsular typing

Pneumococcal molecular serotyping by microarray was conducted on the 16 *lytA* qPCR positive samples (ten of which were culture negative) using DNA extracted from CSF to identify serotypes and to assess the feasibility of conducting molecular serotyping directly from the clinical specimens. Microarray serotyping results were obtained for nine (56%) samples, four of which were culture negative. The following serotypes were identified: 6A, 7B, 7C, 8, 16F, 19A, 23B, 23F, and 44 (n = 1 each). The mean *lytA* Ct values were lower (indicating more pneumococcal DNA) for samples that gave molecular serotyping results compared to those that did not (Ct values 21.5 vs 28.1, P = 0.004, t test). Nine of 13 (69%) samples with Ct values <30 had serotypes identified by microarray compared to zero of three samples with Ct values >30. Two of the culture positive samples also had traditional serotyping performed on the isolates, and results were consistent with microarray. Of the nine pneumococcal serotypes identified, only one (serotype 23F) was covered by PCV10. The 23F was recovered from a patient who was not age eligible for vaccination.

Traditional serogrouping was conducted on three *N. meningitidis* isolates obtained from patients in this study (two from blood cultures and one from CSF). PCR serogrouping was performed on extracted DNA from two culture negative, *sodC* positive CSFs. All five meningococcal samples were serogroup B.

Of the five samples containing *H. influenzae*, two were type b (Hib) and three were non-b. Both patients who tested positive for Hib were under two years old and their Hib vaccinations were up to date for age. One Hib case was a ten week old infant who had received a second vaccine dose two days prior to admission and therefore would not have been fully protected. The other Hib case was a 20 month old child who had received three doses of Hib vaccine; this was classified as a vaccine failure.

## Discussion

In this study, we identified the aetiologic agent in 47 of 266 cases of suspected bacterial meningitis. *S. pneumoniae* and *N. meningitidis* were the two most common bacterial species identified, consistent with a previous meningitis study conducted in Fiji prior to the introduction of PCV10^14^.

Only 18% of meningitis cases had a specific aetiologic agent identified, a rate lower than those obtained from meningitis studies conducted in Turkey (60%)^15^ and Burkina Faso (65% of purulent samples)^16^. These studies used a combination of culture and conventional PCR; the study in Turkey was conducted on patients under 18 years of age whilst the Burkina Faso study was conducted on all ages. The increased detection rate in the Turkey and Burkina Faso studies may be attributable to the high proportion of meningitis cases caused by *N. meningitidis* and *S. pneumoniae* in these countries, and also that the studies were conducted prior to the introduction of PCV and Hib vaccines. Meningitis studies in India (using culture and latex agglutination) and Brazil (using culture and qPCR) identified the causative pathogen in 31% and 25% of CSF samples, respectively^17^,^18^, closer to the results from Fiji. The study in India was conducted on children under five years of age prior to Hib and PCV introduction. The study in Brazil was conducted on patients of all ages prior to PCV introduction and several years after Hib vaccine was introduced. Consequently, there were very few *H. influenzae* positive cases but a relatively high number of pneumococcal cases identified. The proportion of CSF samples from which a causative organism is indentified can vary substantially by population. The low rates of causative organisms identified in Fiji could be due to the fact that two of the main bacterial causes of meningitis, *S. pneumoniae* and Hib, are now vaccinated against, and may also reflect a potential higher contribution of viral meningitis. Differences in lumbar puncture rates and specimen testing procedures can also make it difficult to compare aetiology results between settings.

The use of qPCR significantly improved identification of *S. pneumoniae, N. meningitidis*, and *H. influenzae*, more than doubling the detection rate. These results support the use of qPCR for IB-VPD surveillance, and are consistent with other published studies showing enhanced detection of these pathogens using qPCR^6^,^9^. The current study utilised the DNA extraction and qPCR protocols recommended by the WHO. Although we did not observe any significant differences in the volumes of CSF used for DNA extraction between qPCR positive and negative samples, smaller sample volumes would result in lower DNA yields and could affect sensitivity. Laboratories seeking to maximise sensitivity of pathogen detection may wish to optimise these methods for low-volume samples, for example by lowering elution volumes to concentrate DNA and adding more template DNA to qPCR reactions. It may be also worthwhile to test larger volumes of template DNA for equivocal samples in addition to diluting to check for PCR inhibition.

The use of human blood agar, which is suboptimal for growth of some bacteria including *S. pneumoniae*^19^, may have contributed to the low rate of culture positive samples in this study, and the use of sheep or horse blood is recommended.

Fiji commenced meningitis surveillance in 2012 and joined the WHO Global IB-VPD laboratory network in 2015, which has provided access to technical support and training and an external quality assurance program. It should be noted that the WHO IB-VPD surveillance program only includes children under five years of age, whereas our study included CSF samples collected from patients of all ages. For cost reasons, CSF samples with elevated white blood cell counts (>10 mm^3^; suggestive of meningitis) were prioritised for qPCR testing. The WHO recommends conducting qPCR on all CSF specimens regardless of white blood cell counts, and testing all samples may have further increased IB-VPD case detection. An analysis of IB-VPD surveillance laboratory network data found that samples with lower white blood cell counts were less likely to be qPCR positive, but showed the largest fold increase in detection compared to culture^20^.

We were unable to properly assess the sensitivity of direct antigen testing as the latex testing kits were frequently unavailable during the study period. However, other published studies have found that latex agglutination testing does not significantly improve detection of pathogens in culture negative CSF specimens^5^,^21^. In a multicenter study on detection of pneumococcal meningitis by culture, direct antigen testing, and an immunochromatographic test (ICT) of pneumococcal antigen (Binax NOW), direct antigen testing improved detection of pneumococci when compared to culture at both Asian and African sites, whereas ICT significantly increased detection of pneumococci over culture and direct antigen testing in the Asian but not African sites^22^.

Molecular serotyping by microarray was able to identify pneumococcal serotypes in roughly half of samples using DNA directly extracted from CSF, and this has specific relevance for samples where no isolate is obtained. Sensitivity could potentially be improved by employing an amplification step, such as culture (or bacterial DNA enrichment and whole genome amplification for culture negative specimens) prior to conducting microarray. Molecular serotyping methods that include an amplification step, such as serotype-specific qPCR, may also be useful for pneumococcal serotyping of culture negative specimens. In a meningitis study in Bangladesh, use of sequential multiplex PCR enabled the determination of pneumococcal serotypes in 51/127 (40%) of pneumococcal positive, culture negative CSF samples^23^. A South African study on invasive pneumococcal disease (IPD) used qPCR to identify serotypes in 279/607 (46%) of *lytA* positive blood samples, and noted that the proportion of serotypable samples was higher among samples with lower *lytA* Ct values (<30), consistent with our results^24^.

Although very few pneumococcal serotyping results are available, there were no PCV10 failures identified in this study. PCV10 does not include serotype 19A, which emerged as a major cause of IPD in some populations following introduction of PCV7^25^. Although only a single case of meningitis caused by pneumococcal serotype 19A was identified in our study (in an 18 month old child), ongoing surveillance is warranted to monitor potential shifts in meningitis aetiology including pneumococcal serotype replacement.

Although the total number of *N. meningitis* cases detected was small, it appears to be on the rise (seven cases detected in 2015 compared to two in 2014 and one in 2013), as anecdotally, cases of meningococcemia were uncommon clinically in Fiji. All samples available for serotyping belonged to serogroup B (MenB). In nearby New Zealand, a MenB epidemic occurred predominantly in the Pacific Islander community for many years with substantial associated morbidity and mortality, and was eventually controlled by a strain-specific MenB vaccine developed specifically for this population^26^,^27^. Results from the current study have generated concern about a potential increase in MenB infections in Fiji. Surveillance of meningococcal disease has now been expanded to other regions of Fiji, and available isolates are undergoing whole genome sequencing.

The results from our study highlight the utility of conducting qPCR detection for IB-VPD pathogens. Although data on serotypes/serogroups and molecular epidemiology do not impact clinical care, they are important from a public health perspective, as this information is needed to help aid decision making on immunisation strategies and also to monitor potential changes in serotypes/serogroups following vaccine introduction.

## Additional Information

**How to cite this article**: Dunne, E. M. *et al*. Real-time qPCR improves meningitis pathogen detection in invasive bacterial-vaccine preventable disease surveillance in Fiji. *Sci. Rep.* **6**, 39784; doi: 10.1038/srep39784 (2016).

**Publisher's note:** Springer Nature remains neutral with regard to jurisdictional claims in published maps and institutional affiliations.

## Figures and Tables

**Table 1:** Age and clinical characteristics of patients from whom cerebrospinal fluid samples were obtained (n = 266).

Age			
	N (%)	Median (IQR)	Aetiological agent identified (n)
<1 month	67 (25.2)	8 (2, 19) days	*S. pneumoniae* (1), *N. meningitidis* (1), GBS (1), *K. oxytoca* (1)
1–12 months	106 (39.8)	5.0 (2.0, 7.0) months	*N. meningitidis* (7), S*. pneumoniae* (6), GBS (4*), H. influenzae* (3)
1–5 years	45 (16.9)	1.7 (1.3, 2.3) years	*S. pneumoniae* (7), *N. meningitidis* (3), *E. coli* (3), *H. influenzae* (1), coagulase negative staphylococci (1)
5–18 years	16 (6.0)	10.1 (5.6, 13.3) years	*N. meningitidis* (1), *H. influenzae* (1), *A. baumannii* (1)
adult	32 (12.0)	37.4 (25.7, 53.4) years	*S. pneumoniae* (3), *N. meningitidis* (1), GBS (1)
**Clinical characteristics**			
	**N (%)**	**Aetiological agent identified (n)**	
meningitis	114 (42.3)	*S. pneumoniae* (15), *N. meningitidis* (9), *H. influenzae* (5), GBS (4), *E. coli* (1)	
neonatal sepsis	61 (22.9)	*N. meningitidis* (1)	
sepsis	16 (6.0)	*N. meningitidis* (2), *S. pneumoniae* (1)	
pneumonia	10 (3.7)	GBS (2)	
seizures	9 (3.4)		
fever	7 (2.6)	*N. meningitidis* (1)	
ventriculo-peritoneal shunt	6 (2.2)	*E. coli* (2)	
hydrocephilus	6 (2.2)	coagulase negative staphylococci (1)	
head/brain injury/abnormality	6 (2.2)	*A. baumannii* (1)	
post mortem	5 (1.9)	*K. oxytoca* (1)	
Acute confusion/delirium	3 (1.1)	*S. pneumoniae* (1)	
heart disease/abnormality	3 (1.1)		
Other	20 (7.5)		

IQR = interquartile range; GBS = group B streptococcus. Other includes the following (n ≤ 2 each): asphyxia, low birth weight, congenital syphilis, cellulitis, acute gastroenteritis, jaundice, pulmonary edema, headache, spinal injury, hypoglycemia, limb weakness, chemotherapy.

**Table 2:** Laboratory identification of *S. pneumoniae, N. meningitidis*, and *H. influenzae*.

	Total n	Culture positive n (%)	DAT positive n (%)	Seen by Gram stain n (%)	qPCR positive n (%)	Increased detection due to qPCR (%)*
*S. pneumoniae*	17	7 (41)	6 (54) (6 not tested)	8 (50) (1 not tested)	16 (100) (1 not tested)	200
*N. meningitidis*	13	1 (8)	2 (29) (6 not tested)	3 (25) (1 not tested)	12 (92)	400
*H. influenzae*	5	2 (40)	1 (50) (3 not tested)	1 (20)	5 (100)	250

DAT = direct antigen testing. *Percent increase in identification due to qPCR compared to culture, DAT, and Gram stain combined.
